# *Rcan2* and estradiol independently regulate body weight in female mice

**DOI:** 10.18632/oncotarget.18259

**Published:** 2017-05-29

**Authors:** Ling-Cui Ding, Qian-Qian Gong, Shi-Wei Li, Xiao-Long Fu, Ye-Cheng Jin, Jian Zhang, Jian-Gang Gao, Xiao-Yang Sun

**Affiliations:** ^1^ Institute of Developmental Biology, School of Life Science, Shandong University, Jinan, China

**Keywords:** Rcan2, 17β-estradiol, ovariectomy, body weight regulation, obesity

## Abstract

*Rcan2* increases food intake and plays an important role in the development of age- and diet- induced obesity in male mice. However, in females, wild-type mice grow almost at a similar rate as *Rcan2*^−/−^ mice on normal chow diet from 6 weeks of age. Here we showed that the ability of *Rcan2* to promote weight gain was attenuated by energy expenditure mediated by 17β-estradiol in female mice. Using ovariectomy-operated models, we found that 17β-estradiol deprivation did not alter food intake, but induced more weight gain in wild-type mice than *Rcan2*^−/−^ mice. If wild-type mice ingested equally as *Rcan2*^−/−^ mice, in the same ovarian state they exhibited similar weight changes, but the mice in ovariectomized groups were significantly heavier than the ovarian-intact mice, suggesting that body weight is not only regulated by *Rcan2*, but also by 17β-estradiol. Furthermore, we demonstrated that *Rcan2* and 17β-estradiol independently regulated body weight even on high-fat diets. Therefore, our findings indicate that *Rcan2* and 17β-estradiol regulate body weight through different mechanisms. *Rcan2* increases food intake, whereas 17β-estradiol promotes energy expenditure. These findings provide novel insights into the sexual dimorphism of body weight regulation.

## INTRODUCTION

The regulation of body weight is thought to be sexually dimorphic. Men and postmenopausal women have greater risks of developing obesity and associated co-morbidities, whereas premenopausal women tend to keep a slender body shape [1]. In rodents, the protection against obesity in young females is eliminated by surgical removal of the ovaries (ovariectomy), which can be reversed through the administration of exogenous 17β-estradiol (estradiol) [2–7]. In humans, adiposity in postmenopausal women can be altered by estradiol replacement therapy [8, 9]. Moreover, mice lacking estrogen receptor-α (ERα) [10] or aromatase [11], the enzyme responsible for estrogen biosynthesis, develop obesity and hyperlipidemia. Recently, site-specific reductions of ERα in the brain were reported to result in increased adiposity [12–14]. Accordingly, this evidence highlights that the ovarian hormone estradiol plays an important role in the regulation of body weight in females.

*Rcan2* was originally identified as a thyroid hormone–responsive gene [15]. In mice, two splicing variants that harbor distinct tissue-specific expression patterns have been identified: *Rcan2-3* is expressed predominately in the brain, whereas *Rcan2-1* is expressed not only in the brain but also in other tissues such as heart and skeletal muscle [16]. Recently, in C57BL/6J (B6) mice, a golden animal model of human obesity [17–19], we found that loss of *Rcan2* function ameliorated age- and diet-induced obesity by causing a reduction in food intake [20]. We showed that reduction of food intake, rather than reduced body size, in *Rcan2*^−/−^ mice was a primary effect of *Rcan2* deletion [21]. *Rcan2* expression is most prominent in the ventromedial, dorsomedial and paraventricular hypothalamic nuclei governing energy balance [20]. Using double-mutant (*Lep^ob/ob^ Rcan2*^−/−^) mice, we demonstrated that *Rcan2* and leptin regulate body weight through different pathways [20]. Thus, we hypothesized that *Rcan2* may increase food intake and promote weight gain through a leptin-independent pathway. However, when analyzing the previous results [20], we found that, in males, wild-type (WT) mice progressively gained more weight than *Rcan2*^−/−^ mice with age, regardless of diet composition. In females, when fed a high-fat diet (HFD), WT mice increased body weight more quickly than *Rcan2*^−/−^ mice, and their growth patterns were consistent with those of male mice, whereas on normal chow diet (NCD), the growth curves of WT mice were nearly parallel to those of *Rcan2*^−/−^ mice from 6 weeks of age, a phenomenon showing the sexual dimorphism. Since estradiol plays a critical role in the regulation of females’ body weight, it is supposed that the function of *Rcan2* may be affected by estradiol. Thus, the present study was designed to determine the relationship between *Rcan2* and estradiol in the regulation of body weight in B6 mice.

## RESULTS

### Evaluation of estradiol levels and *Rcan2* expression in mice after surgery

**Figure 1:**
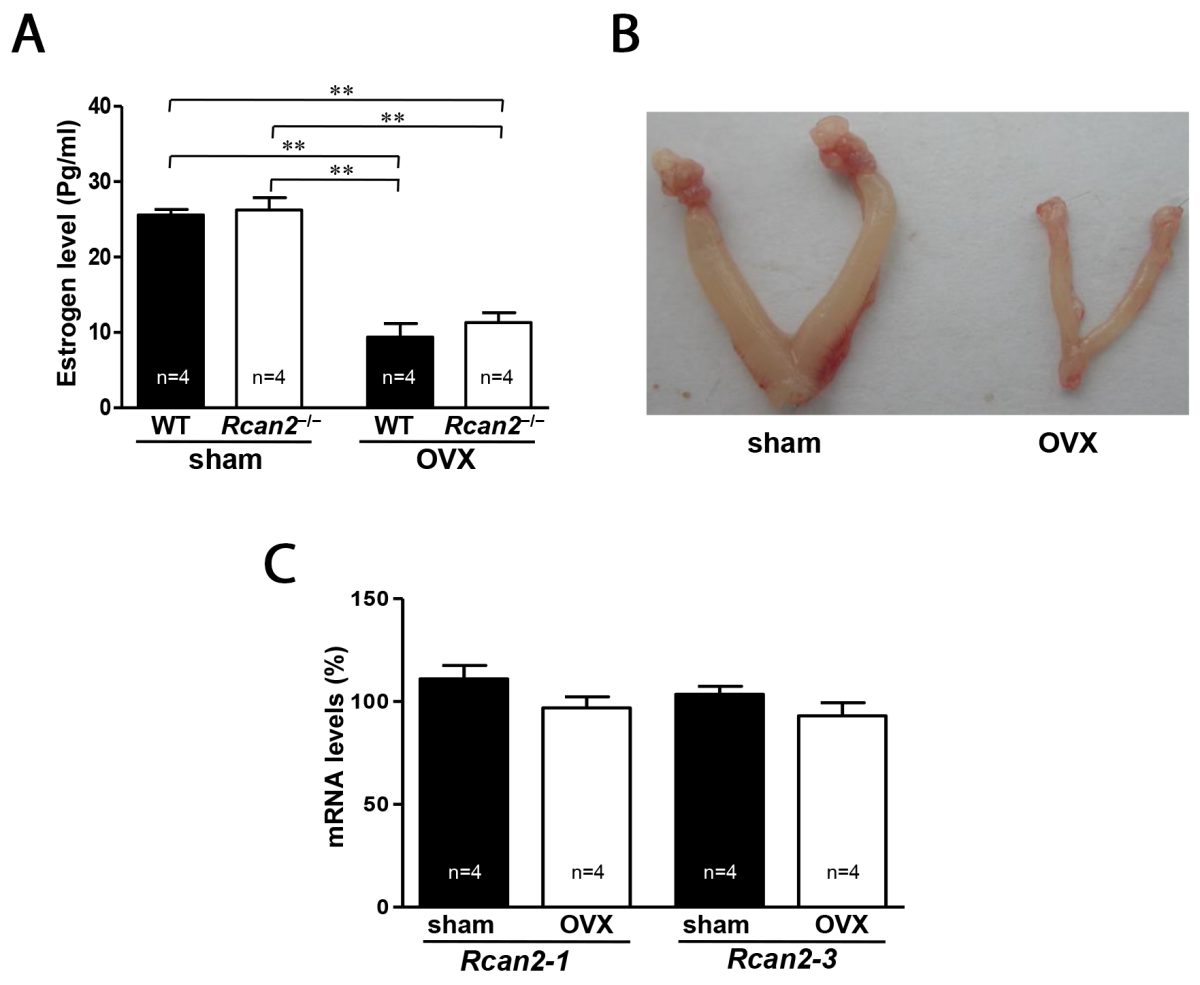
Serum levels of estradiol and expression of *Rcan2* mRNA in OVX mice **(A)** Serum levels of estradiol in sham-operated (sham) and ovariectomized (OVX) mice. Statistics were performed using one-way ANOVA, and individual group differences presented here were measured using Bonferroni's correction. **: *p* <0.01. **(B)** Representative images of uteri in sham mice and OVX mice. **(C)** Expression levels of *Rcan2-1* and *Rcan2-3* mRNA in hypothalamus. Total RNA was isolated from hypothalami of sham and OVX WT mice and subjected to quantitative real-time PCR analysis using primers specific for *Rcan2-1*, *Rcan2-3*, and *β-actin* (internal standard). mRNA expression levels are expressed relative to those of sham WT mice. The number of mice (n) used in the experiments was indicated directly in each panel of figures. All values are given as means ± SEM.

To determine the relationship between *Rcan2* and endogenous estradiol in body weight regulation, we compared the growth of *Rcan2*^−/−^ mice and WT controls in the conditions of estradiol presence or estradiol deprivation by ovariectomy (OVX) surgery. OVX significantly reduced plasma estradiol levels, not only in WT mice but also in *Rcan2*^−/−^ mice (Figure 1A). Two-way analysis of variance (ANOVA) revealed that plasma estradiol levels in mice were only related to the status of the ovary (*p* <0.0001, two-way ANOVA with Bonferroni correction), not *Rcan2* (*p* = 0.39), and there were no interactions between them (*p* = 0.66). Since estradiol regulates the development of the uterus, OVX causes an atrophic uterine phenotype, a phenomenon indicating success of the surgery (Figure 1B). Real-time polymerase chain reaction (PCR) analyses were performed to determine whether *Rcan2* expression was regulated by estradiol. The results revealed that estradiol deprivation by OVX did not alter either the expression of *Rcan2-1*, or that of *Rcan2-3* in WT mice (Figure 1C), suggesting that estradiol did not regulate the expression of *Rcan2*.

### Changes in body weight and body composition after OVX in mice fed NCD

**Figure 2:**
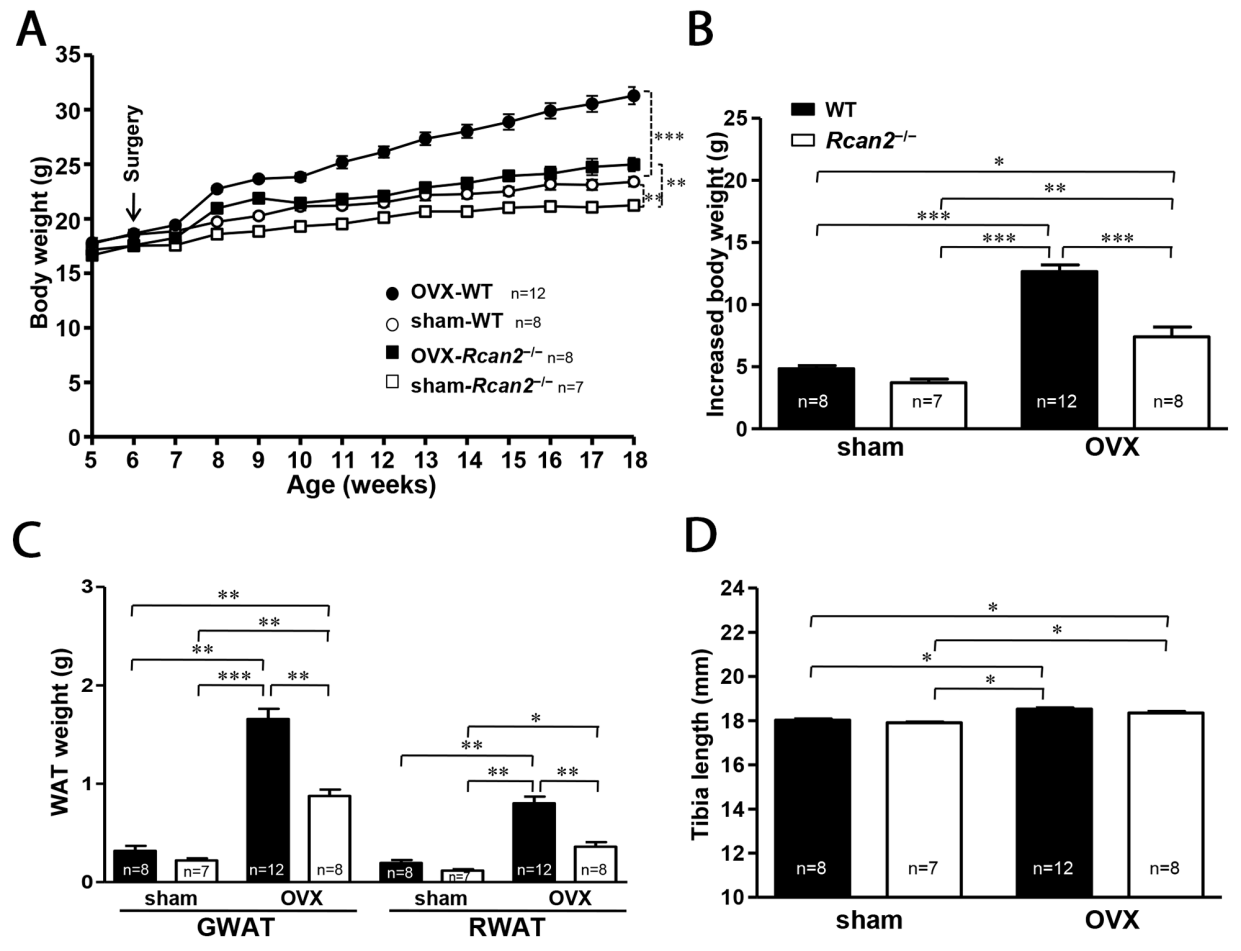
Changes in body weight and adipose tissue deposition in WT and *Rcan2*^−/−^ mice after sham-operation or bilateral ovariectomy fed on a NCD **(A)** Growth curves of sham-operated (sham) females and ovariectomized (OVX) females fed NCD. Arrows show the time of sham-operation or ovariectomy. **(B)** Increased body weight measured from postnatal week 6 to week 18. **(C)** Mean weights of gonadal (GWAT) and retroperitoneal white adipose tissue (RWAT) and **(D)** mean tibia lengths in females at postnatal week 18. The number of mice (n) used in the experiments was indicated directly in each panel of figures. Statistics were performed using one-way ANOVA, and individual group differences presented here were measured using Bonferroni correction. *: *p* <0.05, **: *p* <0.01, ***: p <0.001. All values are given as means ± SEM.

OVX was performed at postnatal week 6. Initial body weights of WT and *Rcan2*^−/−^ mice differed by about 1 g (*p* >0.05) (Figure 2A). Twelve weeks after the surgery, the sham WT female mice weighed an average of 2.2 g more than sham *Rcan2*^−/−^ mice on NCD (23.4 ± 0.4 g in sham WT mice versus 21.2 ± 0.4 g in sham *Rcan2*^−/−^ mice; *p* <0.002) (Figure 2A), while in the OVX groups, OVX WT female mice gained weight more quickly than OVX *Rcan2*^−/−^ female mice. At 18 weeks of age, both WT mice and *Rcan2*^−/−^ mice in OVX group were significantly heavier than the sham counterparts, and the average weight difference increased to 6.3 g (31.3 ± 0.8 g in OVX WT mice versus 25.0 ± 0.6 g in OVX *Rcan2*^−/−^ mice; *p* <0.0001) (Figure 2A). On the other hand, during the period of 12 weeks, OVX WT mice gained significantly more weight than OVX *Rcan2*^−/−^ mice (12.7 ± 0.6 g in OVX WT mice versus 7.4 ± 0.8 g in OVX *Rcan2*^−/−^ mice; *p* <0.0001), while both of them gained more weight than the sham counterparts (4.8 ± 0.3 g in sham WT mice versus 3.7 ± 0.3 g in sham *Rcan2*^−/−^ mice; *p* <0.01) (Figure 2B). Thus, *Rcan2* promotes weight gain *(p* <0.001, two-way ANOVA with Bonferroni correction), and, further, estradiol also affects body weight (*p* < 0.0001). There are interactions between *Rcan2* and estradiol (*p* = 0.003), with WT mice being more affected than *Rcan2*^−/−^ mice. These weight gains were consistent with increased body fat in anatomical analyses. Twelve weeks after OVX, significantly increased body fat was detected not only in WT mice, but also in *Rcan2*^−/−^ mice, while loss of *Rcan2* significantly reduced adipose tissue mass regardless of estradiol level (Figure 2C). Tibia lengths of sham WT mice were similar with those of sham *Rcan2*^−/−^ mice (18.02 ± 0.06 mm in sham WT mice vs. 17.90 ± 0.05 mm in sham *Rcan2*^−/−^ mice; *p* = 0.1), while shorter than the mice in OVX groups (18.52 ± 0.06 mm in OVX WT mice vs. 18.35 ± 0.07 mm in OVX *Rcan2*^−/−^ mice; *p* = 0.07) (Figure 2D). These results further demonstrated that loss of *Rcan2* did not directly affect body size, consistent with our previous result [21]. However, estradiol deprivation was found to significantly increase the tibia length (*p* < 0.001, two-way ANOVA with Bonferroni correction). Taken together, these results suggested that the functions of *Rcan2* and estradiol were independent of each other.

### Changes in food intake and energy expenditure after OVX in mice

**Figure 3:**
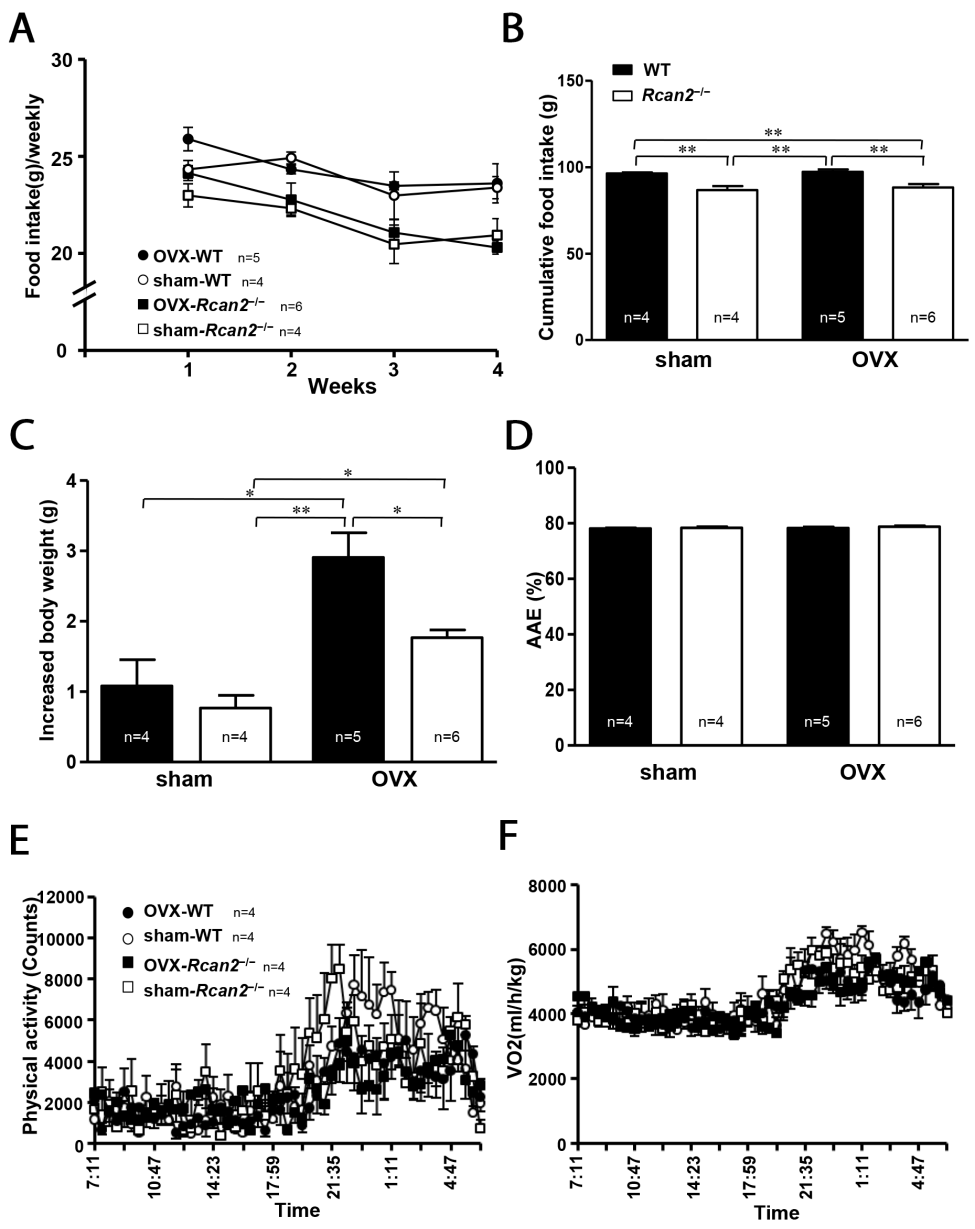
Parameters of food intake and energy expenditure **(A)** Weekly food intake, **(B)** cumulative food intake, and **(C)** increased body weight measured from postnatal week 8 to week 11 in sham-operated (sham) and ovariectomized (OVX) mice fed on a NCD. **(D)** Apparent absorption efficiency (AAE) measured in mice fed the NCD. **(E)** Spontaneous physical activity, and **(F)** spontaneous oxygen consumption in 7-week old sham and OVX mice during the light phase (07:00-19:00 h) and the dark phase (19:00-07:00 h). The number of mice (n) used in the experiments was indicated directly in each panel of figures. Statistics were performed using one-way ANOVA, and individual group differences presented here were measured using Bonferroni's correction. *: *p* <0.05, **: *p* <0.01. All values are given as means ± SEM.

In our previous studies using male mice [20, 21], we demonstrated that body weight differences between *Rcan2*^−/−^ and WT mice were mainly caused by differential food intake, rather than energy expenditure. In the present study, we monitored food intake and weight gain in female mice for a period of 4 weeks from postnatal week 7 to postnatal week 11, so that we could accurately determine the metabolic efficiency by measuring changes in body weight relative to caloric intake over the course of 4 weeks [22]. The amount of chow was assessed twice each week. Weekly food intake results revealed that food intake in OVX mice (both WT mice and *Rcan2*^−/−^ mice) was weakly increased in the first week of measurements compared with the sham counterparts, and then was reduced and maintained at that level of the sham counterparts for the remainder of measurements (Figure 3A). On the other hand, regardless of ovarian removal or not, there is a trend that WT mice consume more food than *Rcan2*^−/−^ mice, although generally there was no statistically significant difference between them (Figure 3A). Cumulative food intake data showed that during this period, sham *Rcan2*^−/−^ mice ingested about 11.1% less food than sham WT controls (86.7 ± 2.6g in sham *Rcan2*^−/−^ mice versus 96.4 ± 0.7g in sham WT mice; *p* <0.01), while OVX *Rcan2*^−/−^ mice ingested about 10.2% less food than OVX WT mice (88.3 ± 2.1g in OVX *Rcan2*^−/−^ mice versus 97.3 ± 1.6g in OVX WT mice; *p* <0.01) (Figure 3B). The similar reduction of food intake in *Rcan2*^−/−^ mice in both OVX and sham groups suggests that *Rcan2* mutation might reduce food intake in a uniform manner regardless of estradiol level. In addition, for the mice with same genotype, no differences in the amount of food intake were found between OVX mice and sham counterparts (Figure 3B). Therefore, differential food intake might be relevant to the *Rcan2* gene, not to estradiol level. Although mice of the same genotype consumed similar amounts of food, OVX mice always gained more weight than the sham counterparts (Figure 3C). Since energy expenditure can be assessed by calculating metabolic efficiency (the ratio between body weight gain and cumulative food intake), in the mice with same genotype, OVX mice have lower energy expenditure than sham mice, suggesting that energy expenditure was affected by estradiol level. However, among the mice with different genotypes, OVX mice did not always gain more weight than sham mice. For examples, OVX *Rcan2*^−/−^ mice gained similar weight as sham WT mice (Figure 3C). Therefore, the above data indicates that body weight changes were not only determined by estradiol (*p* <0.001, two-way ANOVA with Bonferroni correction), but also by *Rcan2* (*p* = 0.014). We also excluded the possibility of malabsorption by calculating apparent absorption efficiency. These results showed neither loss of *Rcan2* nor removal of ovaries had significant effects on food absorption (Figure 3D).

The results of food intake measurements revealed that energy expenditure was affected by estradiol level. To identify the possible mechanism that estradiol affects energy expenditure, we used the TSE Calorimetry Systems to measure the metabolic parameters. One week after surgery, a cohort of 7-week-old mice were selected for the analyses. There was a trend that the mice in the sham groups were more active than the mice in the OVX groups in the dark phase, although there was no statistically significant difference between them (Figure 3E). Within the same surgical group, no difference in physical activity was detected between WT mice and *Rcan2*^−/−^ mice. Consistent with the changes of physical activity, OVX mice tended to consume less oxygen than sham mice during the dark phase (Figure 3F).

### A pair-feeding among OVX mice and sham mice

**Figure 4:**
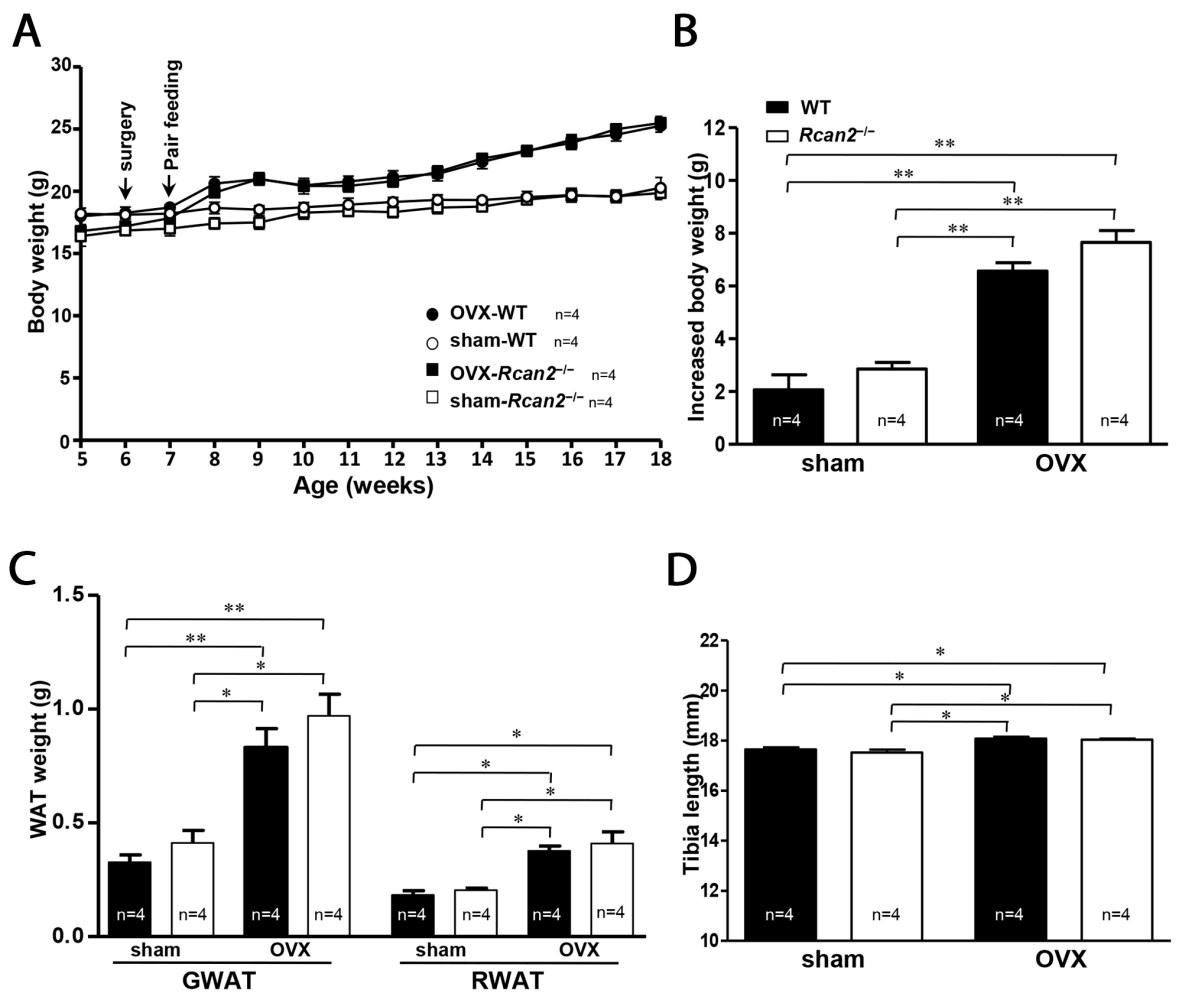
A pair-feeding study between sham-operated and ovariectomized mice on a NCD **(A)** Growth curves, **(B)** increased body weight, **(C)** mean adipose tissue weight (gonadal and retroperitoneal white adipose tissue respectively), and **(D)** mean tibia lengths in sham-operated (sham) mice and ovariectomized (OVX) female mice after 11-week pair-feeding study. Arrows show the time of sham (or OVX) operation and the start point of pair feeding. The number of mice (n) used in the experiments was indicated directly in each panel of figures. Statistics were performed using one-way ANOVA, and individual group differences presented here were measured using Bonferroni correction. *: *p* <0.05, **: *p* <0.01. All values are given as means ± SEM.

Our above results suggested that *Rcan2* gene and estradiol might regulate body weight through different mechanisms. To further confirm our hypothesis, we performed pair-feeding experiments for a period of 11 weeks from postnatal week 7 to week 18. From postnatal week 9, two weeks after commencement of pair-feeding, the body weights of OVX WT mice were similar to those of OVX *Rcan2*^−/−^ mice. This pattern of body weights remained consistent throughout the remainder of the 11-week study (25.3 ± 0.5 g in OVX WT mice versus 25.5 ± 0.5 g in OVX *Rcan2*^−/−^ mice; *p* = 0.74) (Figure 4A), since similar body weights were gained when OVX WT and *Rcan2*^−/−^ mice consumed comparable amounts of food, indicating that their energy expenditures were similar. On the other hand, when sham WT mice consumed the same amount of food as sham *Rcan2*^−/−^ mice, their body weights were also comparable (20.2 ± 0.9 g in sham WT mice versus 19.9 ± 0.5 g in sham *Rcan2*^−/−^ mice; *p* = 0.72) (Figure 4A), suggesting that they also behaved similarly in energy metabolism. After the 11-week pair-feeding, with consuming the similar amount food, the mice in OVX group gained more weight than the mice in sham group (Figure 4A and 4B), further demonstrating that the mice in OVX group have reduced metabolic rate than the mice in sham group. The dissection results revealed that, after the long-term pair-feeding, OVX WT and *Rcan2*^−/−^ mice contained similar amounts of adipose deposits, including gonadal and retroperitoneal white adipose tissue, but the amount of adipose tissue was significantly more than those in the sham groups (Figure 4C). The analyses of tibia lengths showed that, in the same ovarian state, there was no significant difference between WT and *Rcan2*^−/−^ mice, while the tibia lengths of the mice in OVX groups were longer than those in sham groups (Figure 4D). These results were consistent with our above results (Figure 2D), and demonstrated that *Rcan2* and estradiol regulate body weight through different mechanisms.

### Changes in body weight and body composition in OVX mice fed HFD

**Figure 5:**
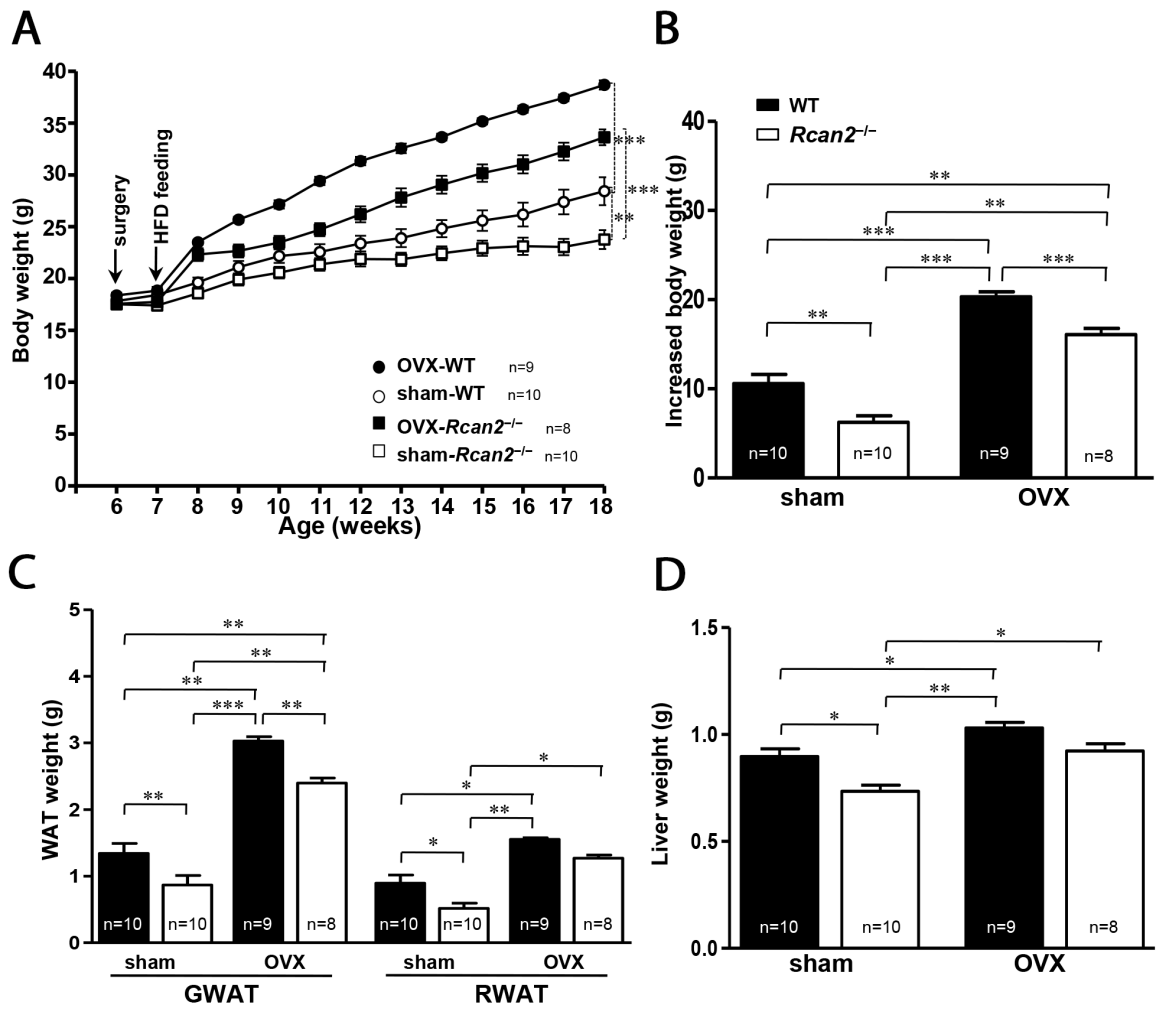
Changes in body and adipose tissue weights of WT and *Rcan2*^−/−^ mice after sham-operation or bilateral ovariectomy fed on a HFD **(A)** Growth curves of sham-operated (sham) females and ovariectomized (OVX) females fed HFD. Arrows indicate the time of sham (or OVX) operation and the start point of high-fat feeding. **(B)** Increased body weight measured from postnatal week 6 to week 18. Mean weights of gonadal (GWAT) and retroperitoneal white adipose tissue (RWAT) **(C)** and liver **(D)** in females at postnatal week 18. The number of mice (n) used in the experiments was indicated directly in each panel of figures. Statistics were performed using one-way ANOVA, and individual group differences presented here were measured using Bonferroni's correction. *: *p* <0.05, **: *p* <0.01, ***: *p* <0.001. All values are given as means ± SEM.

In the prior studies, we reported that WT females gained significantly more weight than *Rcan2*^−/−^ mice on a HFD [20] and it was also reported that HFD could exaggerate OVX-induced obesity [23, 24]. We evaluated the response of loss of *Rcan2* in HFD-fed OVX female mice. After a period of 11 weeks on HFD, both sham WT and *Rcan2*^−/−^ mice were heavier than their counterparts on NCD (see Figure 2A), and the weight difference between them increased to 4.6 g (28.4 ± 1.4 g in sham WT mice versus 23.8 ± 0.9 g in sham *Rcan2*^−/−^ mice; *p* <0.01) (Figure 5A). In the OVX mice, as early as the second week, OVX causes a strikingly faster rate of body weight gain compared with sham mice. However, the weight differences between WT and *Rcan2*^−/−^ mice did not increase greatly at 18 weeks of age (38.7 ± 0.4 g in OVX WT mice versus 33.6 ± 0.8 g in OVX *Rcan2*^−/−^ mice; *p* <0.0001) (Figure 5A), but HFD significantly exaggerated the weight gain in all of the mice (Figure 5B). Thus, even on HFD feeding, OVX significantly promotes weight gain (*p* <0.0001, two-way ANOVA with Bonferroni correction) and *Rcan2* also profoundly affects body weight (*p* <0.0001). However, there were no interactions between *Rcan2* and estradiol in mice on HFD (*p* = 0.84). Anatomical analyses revealed that OVX significantly increased adipose tissue deposition (including gonadal and retroperitoneal white adipose tissue) and liver in female mice, whereas loss of *Rcan2* significantly reduced adipose tissue accumulation, regardless of estradiol levels (Figure 5C and 5D).

## DISCUSSION

In this report we describe the study to determine the relationship between *Rcan2* and estradiol in the regulation of body weight in B6 female mice. First, estradiol deprivation by OVX did not alter *Rcan2* expression in WT mice, but induced more weight gain in WT mice than in *Rcan2*^−/−^ mice. Second, measurement of food intake revealed that WT mice ingested more than *Rcan2*^−/−^ mice regardless of estradiol levels; nevertheless, weight gains during the period of measurement were not only related to food intake, but also to estradiol status. Third, indirect calorimetry analyses showed that physical activity in dark phase tended to decrease in both WT and *Rcan2*^−/−^ mice after OVX. Fourth, pair-feeding experiments showed that if WT mice ingested the same amount of food as *Rcan2*^−/−^ mice in the same ovarian state, they exhibited similar weight changes. However, the weight changes of mice in OVX groups were significantly larger than those in sham groups. Finally, *Rcan2* and estradiol were demonstrated to independently modulate body weight even on obesogenic HFDs. Thus, our data suggest that *Rcan2* and estradiol may regulate body weight through different mechanisms.

Accurately measuring food intake is one of the most important parts in the studies of body weight regulation. However, food intake can be altered by multiple factors, such as stress caused by handlings during the process of food intake measurements. So it is difficult to determine whether the results obtained by the measurements faithfully reflect the actual food intake during the process of mouse growth. In our studies [20, 21], we measured food intake in mice from several weeks to a few months, and also monitored the weight changes during these periods, so that we could analyze the food intake when the weight changes during these periods were consistent with those in the growth curves. Because body weights of the mice have changed in the process of measurements (Figure 3C), we did not normalize food intake to body weight. Actually, we evaluated the metabolic efficiency by measuring the changes in body weights relative to total food intake over the period of measurements [22]. By this method, in the prior study [20], we found that *Rcan2*^−/−^ male mice ingest about 8.5% less NCD and about 10.3% less HFD than WT males; in the present study, sham *Rcan2*^−/−^ female mice consumed about 11.1% less food than sham WT counterparts, and OVX *Rcan2*^−/−^ female mice consumed about 10.2% less food than OVX WT controls (Figure 3B). Thus, we hypothesized that *Rcan2* gene might regulate food intake in a uniform manner, regardless of diet composition, sex, and estradiol levels. On the other hand, in our previous study [21], we found that if we reduced the pup number in the mouse litters, the weight differences between *Rcan2*^−/−^ and WT mice at the time of weaning disappeared, and differences of tibia length also disappeared in adulthood. In the present study, we also showed that there was no significant difference in tibia length between age-matched WT and *Rcan2*^−/−^ females when they were in the same ovarian state. Hence, we concluded that reduction of food intake, rather than reduced body size, in *Rcan2*^−/−^ mice was a primary effect of *Rcan2* mutation. Further, we demonstrated that when WT male mice were only allowed to ingest same amount of HFD as *Rcan2*^−/−^ males, the weight gain and body fat mass in WT mice were not different with those in *Rcan2*^−/−^ mice [21]. In the present study, if WT female mice consumed equally with *Rcan2*^−/−^ mice in the same ovarian state, their body weight trajectories were nearly identical (Figure 4A). These data suggest that, the tremendous difference in body weight and body composition between WT mice and *Rcan2*^−/−^ mice may solely result from differential food intake. Therefore, our findings indicate that *Rcan2* increases food intake in a uniform manner and promotes weight gain, regardless of food composition, sex, and estradiol levels.

Menopause is a critical period in a woman's life, which is characterized by reduced secretion of ovarian hormone, especially estradiol, due to age. Removal of ovarian hormones in rodents by OVX mimics menopause in women, increasing the susceptibility to obesity and its associated comorbidities. It was initially reported that estradiol exerts a tonic inhibitory effect on meal size and daily food intake throughout the ovarian cycle, and a cyclic inhibitory effect during the periovulatory phase [25, 26]. Estradiol deficiency and OVX have been reported to increase food intake and fat accumulation in rodents and human [26–30]. However, hyperphagia in the estradiol-deficient rats has also been reported to be transient, and obesity was maintained in these rats by decreased spontaneous physical activity and thermogenesis [7, 31–33]. In B6 mice, ovariectomy-induced increases in weight gain were recently demonstrated to be caused by a decrease in energy expenditure, rather than an increase of food intake [34–36]. In menopausal women, decreased energy expenditure was also suggested to contribute to increased visceral adiposity [37]. Similar conclusions were obtained in ERα knock-out mice, in which increased adiposity was attributed primarily to decreased energy expenditure, and overeating did not appear to be essential [38–41].

In the present study, our results showed that, both in WT and *Rcan2*^−/−^ mice, withdrawal of estradiol after bilateral OVX led to a slight increase in food intake in the first week of measurement, and, thereafter, intake was declined and remained at the levels of sham controls (Figure 3A). On the whole, estradiol deprivation did not significantly alter the amount of cumulative food intake during the period of measurement (Figure 3B). These changing patterns of food intake were consistent with the early findings [7, 31–36], but there was a significant difference in the total amount of food intake between WT and *Rcan2*^−/−^ mice (Figure 3B). Since withdrawal of estradiol after OVX did not alter the total amount of food intake, but significantly increased body weight in mice (Figure 3C), it indicated that estradiol deprivation resulted in reduced metabolic rate in mice. We further verified this assumption by the pair-feeding experiments. In the same ovarian state, when WT mice were only allowed to ingest same amount of food as *Rcan2*^−/−^ mice, the weight gain and body fat mass in WT mice were similar with those in *Rcan2*^−/−^ mice. However, the mice in OVX group were significantly heavier than those in sham group (Figure 4A and 4B). These data suggest that body weight was not only affected by food intake, but also by estradiol levels. Further, indirect calorimetry analyses were performed to determine how estradiol affects energy expenditure. Our result showed that OVX mice tended to manifest reduced physical activity and oxygen consumption than sham mice in the dark phase, but this difference was not statistically significant (Figure 3E and 3F). These results were a little different with studies in the literatures [33, 35, 42], which showed loss of ovarian function in mice resulted in decreased activity in the dark phase. The reason for the inconsistency is unknown in this study, but it can possibly be attributed to the TSE Calorimetry Systems which include eight chambers used in the present study. A key point may be that there is only one analyzer to measure the metabolic parameters in a single chamber at a time. So each mouse in the system is not always being monitored in the whole time. According to the discussion in the literature [43], each animal in such system is measured for just 1 min every 24 min (4.1% of the time). On the other hand, the activities of the mice monitored by such systems are not continuous. There is a possibility that the movement of the mouse is not synchronized with the measurement of the system, so the final measurement results may not absolutely reflect the actual activities of the mice. Thus, to clarify the mechanisms of estradiol on body weight regulation, it is necessary for us to apply a system in future studies that could monitor energy expenditure more accurately.

It is known that male mice are more prone to be obese than female mice when fed a HFD, and withdrawal of estradiol by OVX eliminates the protection of female mice from becoming obese [23]. In our previous study [20], we showed that WT females gained weight more quickly than *Rcan2*^−/−^ females when fed a HFD. In fact, both WT and *Rcan2*^−/−^ mice on HFD weighed more than their counterparts on NCD, indicating that all of them have a greater propensity of gaining body weight when exposed to HFD, regardless of their genotypes. In the present study, we also showed that both sham WT and *Rcan2*^−/−^ mice on HFD gained more weight than their counterparts on NCD (Figure 2A and 5A), even though the composition of the diets used in this study was different from those in the previous study [20]. These findings agree with previous studies that HFD induces weight gain in B6 female mice [23, 44]. On the other hand, as described above, withdrawal of estradiol by OVX also exerted a significant effect on weight gain. The interaction between estradiol deprivation and HFD feeding seems to significantly aggravate their effect on body weight in mice, leading to profound weight gain and fat pad accumulation (Figure 5). These effects were not only seen in WT mice, but also in *Rcan2*^−/−^ mice, suggesting that these effects were independent of mouse genotypes. Recently, Ludgero-Correia and coworkers reported that the combination of OVX and HFD in B6 mice led to increased adiposity [24]. However, in their study, OVX alone did not lead to obesity, but exerted a major influence on obesity induction in the OVX females on HFD.

In conclusion, our results indicate that *Rcan2* and estradiol might regulate body weight through different mechanisms in female mice. *Rcan2* increases food intake and promotes weight gain, while estradiol increases energy expenditure possibly by regulating physical activity. The interplay of *Rcan2* and estradiol on body weight might contribute to the nearly parallel growth curves between WT female mice and *Rcan2*^−/−^ female mice on NCD. In addition, it seems that HFD feeding alone also promotes weight gain, which is independent of *Rcan2* gene and estradiol levels. These findings provide novel insights into sexual dimorphism of body weight regulation and are likely to have important implications for studies on obesity in humans. Future work is needed to determine the mechanisms that *Rcan2* increases food intake and that estradiol mediates energy expenditure.

## MATERIALS AND METHODS

### Animals

The *Rcan2*^+/−^ mouse (RBRC04891) was imported from the RIKEN BioResource Center (Tokyo, Japan). WT (n=55) and *Rcan2*^−/−^ female mice (n=52) were obtained by mating *Rcan2*^+/−^ mice in this study. Parental heterozygous mice used in this study were derived by backcrossing original *Rcan2*^+/−^ to B6 mice for more than 10 generations. Pup genotypes were determined by PCR [20].

Mice were maintained in individually ventilated cage systems at Shandong University School of Life Science, under conditions of 22–24°C, a 12 h light/12 h dark cycle, *ad libitum* access to NCD (4% fat, 3,650 kcal/kg, SLACOM^®^ Mouse Breeder, Pu Lu Teng Biotechnology Co., Shanghai, China), and distilled water, unless noted otherwise. Body weight was measured at 16:00~18:00 once per week using a digital electronic balance.

All animal experimental procedures were approved by Ethics Committee of Shandong University. Animal management was performed strictly in accordance with the standards of the Animal Ethics Committee of Shandong University (Permit Number: ECAESDUSM 20123019).

### Ovariectomy or sham surgery

At 6 weeks of age, mice were anesthetized with intraperitoneal pentobarbital (5 μg/g) and randomly subjected to ovariectomy (OVX) (29 WT mice and 26 *Rcan2*^−/−^ mice) or sham operation (26 WT mice and 26 *Rcan2*^−/−^ mice). Briefly, OVX was performed by the bilateral dorsal abdominal approach so that the ovary and the oviduct could be rapidly removed. Each ovary was cauterized and excised at the tip of the uterine horn. In the sham procedure, a similar incision was made and the ovary was visualized, but no tissue was removed. After the surgery, mice were randomly divided into groups according to the experimental design.

The success of the OVX procedure was confirmed at the end of the study by measuring plasma 17β-estradiol concentration. As 17β-estradiol influences uterine development, atrophy of the uterus was also used as an indicator of the success of the OVX surgery. The data of OVX mice with nonatrophic uteri were excluded in the later analyses.

One week after OVX or sham operation, mice (n = 8−10 per group) were provided with HFD (60% fat, 5,240 kcal/kg, D12492, Research Diets, New Brunswick, NJ) for the subsequent 11 weeks. During treatment, fresh rations were distributed every 2 days.

### Serum 17β-estradiol analyses

The blood samples were incubated at 37°C for 1 h. Thereafter, the samples were centrifuged at 3,000 r/min for 30 min at room temperature and the supernatant was collected and stored at –80°C until processed for 17β-estradiol analysis. Serum 17β-estradiol levels were determined by using enzyme-linked immunosorbent assay (ELISA) kits (Uscn Life Science, Wuhan, China) according to the manufacturer's instructions.

### Pair-feeding studies

One week after the surgery, four mice of the same genotypes were housed in individual cages. Pair-feeding was accomplished by measuring the food intake of the *ad libitum*-fed sham *Rcan2*^−/−^ female mice every 24 hours and providing the same amount of food to pair-fed sham WT mice or OVX mice (both *Rcan2*^−/−^ mice and WT mice). Body weight was recorded every week. After 11 weeks of pair-feeding experiments, mice were anesthetized and fat pads and livers were removed and weighed using a digital electronic balance.

### Food consumption measurements

Two weeks after surgery, mice (n = 4–6 in each group) were housed in individual cages with a steel screen. The consumption of NCD and body weight were then measured for a period of 4 weeks. Food consumption was estimated by subtracting the amount of food left in the cage dispenser and the amount of food spilled from the initial food weight. Mouse droppings were also collected, dried for 2 days, and weighed. The apparent absorption efficiency was calculated as described [20].

### Energy expenditure analyses

At 7 weeks of age, 1 week after the surgery, subsets of mice (n = 4 per group) were placed in metabolic chambers (TSE Calorimetry Systems, Chesterfield, MO) to measure oxygen consumption and physical activity for 48 h. The mice were acclimatized for 24 h before measurements commenced.

### Body composition measurement

At age of 18 weeks, animals were anesthetized with pentobarbital. Blood samples were obtained by puncturing the right cardiac ventricle. Hypothalami were immediately removed and placed in liquid nitrogen, and then stored at –80°C. Liver and white adipose tissue deposits (retroperitoneal and gonadal) were dissected and weighed using a digital electronic balance. The left tibia from each animal was isolated and measured using a vernier caliper.

### Real-time PCR

Total RNA was extracted from hypothalamus using TRIzol reagent (Invitrogen, Carlsbad, CA). Complementary DNA (cDNA) was synthesized from total RNA with a RevertAid first-strand cDNA synthesis kit (Thermo Scientific, Waltham, MA). Real-time PCR was carried out using the SYBR Green Premix Ex Taq (TaKaRa Biotechnology, Dalian, China) and performed in the Light Cycler 480 System (Roche Applied Science, Indianapolis, IN). Relative levels of mRNA were normalized against *β-actin*, then averaged and expressed as means ± SEM. Primers used for all PCR experiments were described previously [20].

### Statistical analysis

Data were expressed as means ± SEM, and were considered significant at *p* <0.05. Statistical analysis of the data was performed using two-tailed distribution Student's t-test, one-way or two-way ANOVA followed by Bonferroni's multiple comparison correction using GraphPad Prism 5.01 (GraphPad Software, La Jolla, CA, USA).
